# Attachment and Mentalizing Abilities in Patients with Inflammatory Bowel Disease

**DOI:** 10.1155/2019/7847123

**Published:** 2019-12-05

**Authors:** Alessandro Agostini, Eleonora Scaioli, Andrea Belluzzi, Massimo Campieri

**Affiliations:** ^1^Department of Experimental, Diagnostic and Specialty Medicine DIMES St.Orsola-Malpighi Hospital, University of Bologna, Bologna, Italy; ^2^Department of Medical and Surgical Sciences DIMEC St.Orsola-Malpighi Hospital, University of Bologna, Bologna, Italy

## Abstract

**Background:**

Inflammatory bowel diseases (IBD) are associated with stress, poor quality of life, and attachment insecurity. Mentalization is the human ability to perceive and reason about feelings and psychological dispositions of one's self and others. The chronic disorders are believed to affect patients' mentalizing abilities and to determine a shift towards attachment insecurity in patients affected. In this study, the attachment dimensions and mentalization were assessed in IBD patients and healthy controls. Further knowledge about the interplay among IBD, mentalization, and attachment might shed more light into the psychopathological mechanisms leading to insecurity and vulnerability to stress in IBD.

**Methods:**

A group of 96 IBD patients and 102 healthy controls completed the attachment style questionnaire (ASQ), the reflective functioning questionnaire (RFQ), and the Eyes test, a performance-based measure of mentalization.

**Results:**

Compared to controls, IBD patients have shown more pronounced attachment anxiety and lower scores in the Eyes test. Disease activity was negatively correlated with the Eyes test scores.

**Conclusion:**

These findings have suggested a plausible impact of IBD on mentalization abilities and have provided new insights into the interplay between IBD, deficits in mentalization, and attachment insecurity. IBD patients are highly vulnerable to disease-related stress that may promote impairments in mentalization. Low mentalization might play a central role in the development of attachment insecurity and emotional disturbances in IBD. The present study's results might open new scenarios for psychodynamic approaches to the treatment of the emotional disturbances in IBD based on attachment and mentalization theory.

## 1. Introduction

Inflammatory bowel diseases (IBD) are chronic intestinal disorders including Crohn's disease (CD) and ulcerative colitis (UC). IBD are associated with stress [1–4], poor quality of life [5], stigma [6], and psychopathological disorders [7]. In particular, IBD have a considerable negative impact on emotions [8], social interactions [9], and close interpersonal relationships [10–12].

Traditionally, IBD have been associated with alexithymia, the deficit in the cognitive processing of emotions [13], while recently, in IBD patients, functional magnetic resonance imaging (fMRI) studies have shown alterations in the neural processing of stress [1, 2] and emotions [8]. The negative impact of IBD on the close interpersonal relationships has been investigated in the light of the attachment theory [5, 10, 11]. This developmental theory states that infants, through the interactions with caregivers, develop cognitive schemas of interpersonal relationships named attachment styles that guide affects and behavior during the life span [14, 15]. If infants experience their caregivers as reliably available and responsive, a secure attachment style develops, characterized by effective regulation of stress and affects. In contrast, if the caregivers are inconsistently responsive, unavailable, or abusing, an insecure attachment style develops characterized by two fundamental dimensions named attachment anxiety and attachment avoidance [14, 15]. Although the attachment style is believed to be a stable personality trait, IBD and other chronic illnesses represent adverse conditions promoting a shift towards attachment insecurity in patients affected [5, 16–19]. The attachment theorists have named mentalization the human ability to perceive, represent, and reason about the emotions, intentions, beliefs, and psychological dispositions of one's self and others and have operationalized this concept as reflective functioning. The relation of alexithymic traits to mentalization has been investigated in previous studies in nonclinical samples [20]. The foundation for mentalizing is rooted in early infant caregiver attachment relationships and matures over the course of development with continued interpersonal interactions [21–23]. Failure in mentalizing functions is thought to promote vulnerability to stress and psychological disorders [24]. It has been hypothesized that the course of the chronic disorders might have negative effects on patients' mentalizing abilities [5, 23]. Impairments in mentalization, in turn, are believed to heighten feelings of insecurity that play a fundamental role in the development of attachment insecurity [21, 22].

Although psychosocial factors and attachment dimensions have been evaluated in IBD patients [4, 11, 25], the mentalizing abilities and the relationships between mentalization and attachment security still need to be investigated in IBD.

In the present exploratory study, the attachment dimensions and mentalization were assessed in a group of IBD outpatients and healthy controls. Further knowledge about the complex interplay among IBD, mentalization, and attachment might shed more light into the psychopathological mechanisms leading to insecurity and vulnerability to stress and psychological disorders in IBD and might pave the way to mentalization-based approaches for psychotherapeutic interventions in distressed IBD patients.

## 2. Methods

This cross-sectional study was conducted in the IBD Unit of St.Orsola-Malpighi University Hospital in Bologna. The local ethic committee approved the study, and all participants signed an informed consent. All patients were clinically evaluated by fully trained physicians of the IBD unit. Moreover, trained psychologists of the department of psychology of the University of Bologna interviewed the participants and administered the psychometric questionnaires.

### 2.1. Population

#### 2.1.1. IBD Patients

Eligible consecutive outpatients attending the IBD unit were evaluated during the routinely control visits and invited to participate in the study. Patients were recruited when they met the inclusion and exclusion criteria and, after acceptance, were instructed by the psychologists to fill out the questionnaires. The following parameters were collected at recruitment: age, sex, education, disease duration, disease activity, previous surgery, use of biologics, and current treatments. The disease activity was measured by means of Crohn's disease activity index (CDAI) [26] for CD (≤150 = remission, 151‐219 = mild activity, 220‐250 = moderate activity, and >450 = severe activity) and the Partial Mayo Score (PMS) [27] for UC (<2 = remission, 2‐4 = mild activity, 5‐7 = moderate activity, and >7 = severe activity).

*Inclusion criteria*: the inclusion criteria were as follows: age > 18 years and <70 years, IBD diagnosis, and IBD diagnosed from at least one year.

*Exclusion criteria*: the exclusion criteria were as follows: nonnative Italian speaker and presence of psychiatric disorders. The battery of questionnaires included the validated Italian translation of the Eyes test [28] (see below). For this test, in order to avoid confounding factors, native speakers are recommended [29]. A semistructured interview based on DSM V [30] was used for the screening of psychiatric disorders.

#### 2.1.2. Healthy Controls

A group of healthy subjects were recruited with advisements and interviewed by the psychologists.

The inclusion criteria were as follows: age > 18 years and <70 years and native Italian speaker. The exclusion criteria were as follows: presence of acute or chronic health disorders including psychiatric disorders and irritable bowel syndrome and current treatment with drugs.

### 2.2. Psychometric Questionnaires

#### 2.2.1. ASQ

The attachment style questionnaire (ASQ) [31] is a self-report questionnaire containing 40 items designed to assess attachment dimensions. Each item corresponds to a statement, and participants must rate if they agree or disagree with it using a six-point Likert scale in which 1 corresponds to “totally disagree” and 6 to “totally agree.” The ASQ contains 5 subscales: (1) Confidence, describing secure attachment, (2) Discomfort with closeness and (3) Relationships as secondary, both assessing attachment avoidance, (4) Need for approval, and (5) Preoccupation with relationships, both assessing attachment anxiety.

#### 2.2.2. RFQ

The reflective functioning questionnaire (RFQ) brief version [32, 33] is a self-report, eight-item questionnaire designed to assess mentalization abilities. The items are statements such as “Sometimes I do things without really knowing why” that are scored by participants using a 7-point Likert scale, ranging from 1, corresponding to “completely disagree,” to 7, corresponding to “completely agree.” The RFQ contains two subscales labelled as certainty (RFQc) and uncertainty (RFQu) about mental states. Low scores in RFQc describe people characterized by rigid certainty about the mental states they attribute to themselves and others (low mentalization), while high scores designate people who typically engage in thinking about how mental states influence behaviors (high mentalization). Conversely, high scores on RFQu designate people with difficulties in mentalization, while low scores in RFQu reflect “acknowledgment of the opaqueness of one's own mental states and that of others, typical of genuine mentalizing” [32, 33].

### 2.3. Reading the Mind in the Eyes Test (Eyes Test)

**Figure 1:**
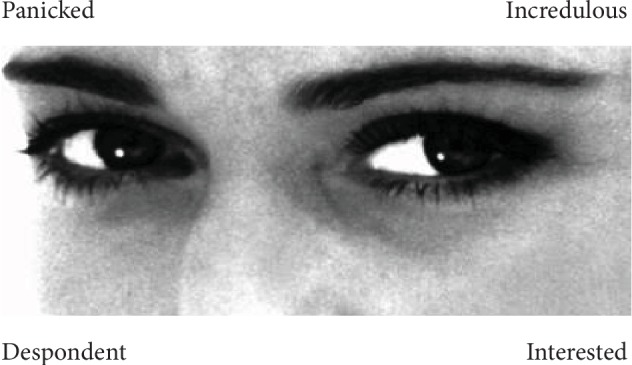
Example of one of the 36 photos of the Eyes test.

The Eyes test [29] was developed to provide a performance-based measure of mentalization. It consists of 36 grey-scale photos (Figure 1) of people taken from magazines, cropped and rescaled in order to show only the area around the eyes. Each photo is surrounded by four adjectives describing four mental states. The participants are instructed to choose, among the four words, the adjective that best describes what the person in the photo is feeling. Among the four items, only one is deemed correct. Indeed, in the initial psychometric study, an independent panel of judges underwent the test and provided consensus about the correct answers. Self-report questionnaires, such as the RFQ, reflect self-perception and evaluation of mentalization. Conversely, the Eyes test is an advanced task involving mental state attribution and complex facial emotion recognition from photographs providing a measure of the mentalization abilities independent from self-perception. Finally, it has been well established that the Eyes test reveals a sex difference, with males scoring significantly lower than females [34]. In the present study, the Italian validated version of the test was used. The reliability and construct validity of the Italian version were assessed in 200 ungraduated Italian students (age 18-32 years) of both sexes in a previous study. This study has shown and confirmed the validity of the Eyes test, good internal consistency, and test-retest stability for the Italian version of the Eyes test. Finally, female scored significantly higher than male and the Eyes test scores were unrelated to social desirability [28].

### 2.4. Statistics

Statistical analyses were conducted using SPSS. The Mann-Whitney *U* test was used for comparisons between IBD patients and HC, between CD and UC patients, and between male and female. The chi-squared test was used for categorical variables. Correlation coefficients were calculated to test for associations between the following parameters: ASQ (1) Confidence, (2) Discomfort with closeness and (3) Relationships as secondary, (4) Need for approval, and (5) Preoccupation with relationships; RFQ (6) certainty, (7) uncertainty, (8) disease activity, (9) disease duration, and (10) age at diagnosis. Pearson's *r* and Spearman's rho were used for correlations.

## 3. Results

### 3.1. IBD Patients

The sample of IBD patients consisted of 96 subjects (64 with CD and 32 with UC).

The females were 48 and the mean age was 39.64 years (±14.06), ranging from 18 to 69 years. The mean IBD duration was 12.39 years (±12.39) ranging from 1 to 48 years. Based on CDAI and PMS scores, 55 patients were in clinical remission, 22 patients had mild disease activity, and 19 had moderate disease activity. Forty patients were currently in treatment with biologics (adalimumab, infliximab, and vedolizumab) while 29 patients had maintenance treatment with 5-aminosalicylic acid agents (5-ASA). Three patients had azathioprine, and 5 were treated with corticosteroids. Finally, 21 patients were previously treated with surgery (Table 1).

### 3.2. Healthy Controls

A group of 102 healthy subjects (50 females) was enrolled as healthy controls for the study. The sociodemographic characteristics of this group are resumed in Table 1.

### 3.3. Comparisons

When we compared the scores obtained in the attachment dimensions between IBD patients and healthy controls, significant differences emerged in the dimension ASQ Preoccupation with relationships describing attachment anxiety. IBD patients had higher scores in this ASQ attachment anxiety measure (*p* < .05).

The two study groups had similar scores in the self-report measures of mentalization (RFQ). Conversely, compared to controls, IBD patients had lower scores (*p* < .001) in the Eyes test. As for the Eyes test scores, in the control group, the female had a significantly higher scores compared to the male (*p* = .012). This typical sex difference was not found in the IBD patient group (Table 1).

Finally, there were no differences between CD and UC patients in the measure of mentalization and attachment but ASQ Preoccupation with relationships. In this dimension, compared to CD patients (27.05 ± 5.79), UC patients (32.19 ± 7.02) had higher scores (*p* = .001).

### 3.4. Correlations

In both IBD and healthy control groups, there were no correlations between the standard test (Eyes test) and self-report measures (RFQ) of mentalization. Moreover, there was no correlation between the Eyes test and the ASQ scores in both IBD patient and healthy control groups, but ASQ Relationships as secondary (*r* = .295, *p* = .004) in the IBD group (Tables 2 and 3).

On the other hand, in the control group, the RFQc scores were negatively correlated with ASQ Discomfort with closeness (*r* = ‐.275, *p* = .005), ASQ Need for approval (*r* = ‐.234, *p* = .018), and ASQ Preoccupation with relationships (*r* = .377, *p* < .001). Furthermore, the RFQu scores were positively correlated with ASQ Need for approval (*r* = .252, *p* = .011) and ASQ Preoccupation with relationships (*r* = .300, *p* = .002) (Table 2).

In the IBD sample, positive correlations were found between RFQu and ASQ Need for approval (*r* = .243, *p* = .017) and ASQ Preoccupation with relationships (*r* = .267, *p* = .009). Moreover, negative correlations were found between the Eyes test scores and disease activity (*r* = ‐.327, *p* = .001). Finally, there were no correlations between disease duration and the psychometric measures but ASQ Preoccupation with relationships (*r* = ‐.217, *p* = .034) (Table 3). Similarly, of all the dimensions, only ASQ Preoccupation with relationships was correlated with age at diagnosis.

## 4. Discussion

In this study, the mentalizing abilities, as measured by the RFQ and the Eyes test, and the attachment dimensions were investigated in a group of IBD outpatients and healthy controls. Compared to controls, IBD patients have shown more pronounced attachment anxiety and lower scores in the psychometric measures of mentalization.

Attachment insecurity, characterized by attachment anxiety and avoidance, has been found in previous studies in IBD patients [3, 8] and patients suffering from chronic disorders [16, 18, 19, 35, 36]. These studies have suggested that the health disorders could represent adversities promoting attachment insecurity in patients [37]. Although the cross-sectional design of this study does not permit causal inferences about the relationship between IBD and attachment, the present findings reinforce the hypothesis that the onset and course of IBD may contribute to the development of attachment insecurity.

**Figure 2:**
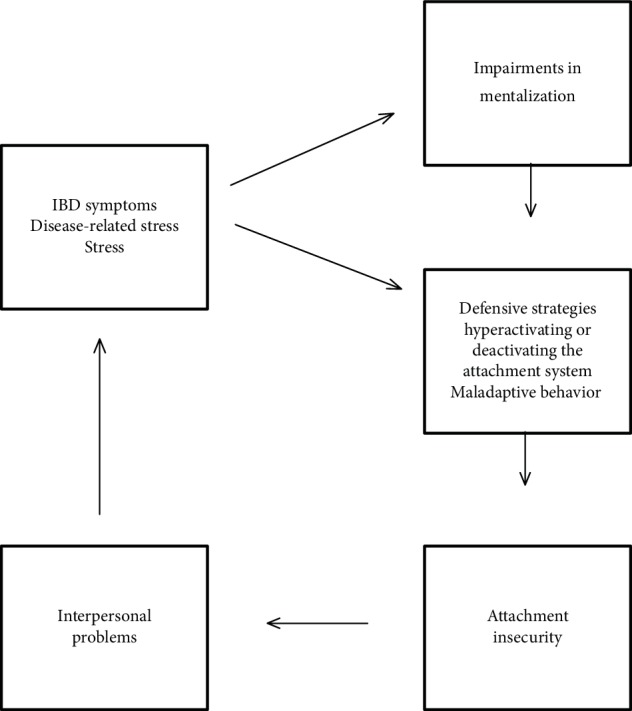


The correlation analyses, together with the comparisons of the mentalization measures between IBD patients and controls, have provided new insights into the complex interplay between illness, deficits in mentalization, and attachment insecurity. The negative correlation found between the Eyes test scores and disease activity and the absence of the sex difference in the Eyes test results in IBD patients have suggested that IBD may affect mentalization abilities in patients affected. Consistent with previous studies [28], there were no correlations between the Eyes test and self-report measures of mentalization. On the other hand, the correlations found between the ASQ and RFQ subscales suggested that the perception of the attachment relationships are likely to be conditioned by the perception of mentalization [38]. Mentalization refers to the mental processes essential to recognize and reflect on the feelings, motivations, beliefs of the self, and others. The chronic illness and disease-related prolonged stress are believed to affect the mentalizing processes [23]. IBD patients are highly vulnerable to disease-related stress that, in turn, may promote impairments in mentalization. Indeed, IBD are chronic diseases that begin at a young age (around the third decade) and significantly affects the quality of life of patients [5, 39]. The chronic course and symptoms of IBD, mainly diarrhea and abdominal pain, promote in patient experiences of embarrassment and a sense of vulnerability and inability. Moreover, IBD relapses are unpredictable and may require hospitalization or even surgery. IBD symptoms can be seen as an attack from within to the capacity to recognize and reflect on the inner mental states and to the capacity to reflect on one's own bodily experiences and their relationships with emotions and feelings [40]. Impairments in mentalization lead to behaviors that exacerbate problems in interpersonal relationships that, in turn, promote attachment insecurity (Figure 2). Indeed, in the context of low mentalization, when faced with disease symptoms and disease-related stress, IBD patients, in the attempt to cope with their illness, may use defensive strategies that hyperactivate or deactivate the attachment system. Patients hyperactivating the attachment system exhibit efforts to seek proximity through demanding, clinging, and claiming behaviors. Conversely, avoidant patients tend to deactivate the attachment system and to cope with autonomy, cognitive distancing from emotions, and denial of distress. The excessive use of these defensive strategies lead to behaviors that further perpetuate interpersonal problems [38]. Indeed, on the long run, patients fail to find social support and understanding through the demanding or avoidant behaviors. These behaviors are often experienced by others as excessive and elicit in them resentment and frustration. Therefore, patients may receive confirmation of their worst fear that they are misunderstood and rejected by others. As a result, patients experience increasing levels of attachment insecurity and further stress that, in turn, exacerbates already existing impairments in mentalizing [18, 23] (Figure 2). The present study is preliminary; nevertheless, its results represent a first step towards the understanding of the dynamics of this vicious circle in the context of IBD patients.

Several limits of this study need to be addressed. First, the enrolled patients were inhomogeneous in terms of duration of the disease, current and previous therapies. The present results need to be replicated in larger samples of patients. Moreover, the present study is correlational and does not permit causal inference. Further longitudinal studies might attempt to clarify the complex relationships between the chronic disorder, attachment insecurity, and mentalization abilities. Five enrolled patients were taking corticosteroids. These drugs may have influenced the mood of patients and affected the psychometric results. Psychological stress and quality of life measures were not collected in participants. Further studies investigating the relationships between stress and mentalization are warranted. Finally, the control visits might represent stressors for IBD patients, and this might have influenced the psychometric measures of mentalization in patients.

In conclusion, to our knowledge, this is the first study investigating the mentalizing abilities and the attachment style in patients with IBD. The results of this study represent a first attempt to thoroughly understand the complex interrelationship between chronic disease, mentalization, and insecure attachment. This study has shown impairments in mentalization in IBD patients and has suggested that low mentalization may play a central role in the development of emotional disturbances in IBD. Improved knowledge of these psychological dynamics may provide new insights into the determinants of psychological stress and deterioration in quality of life in patients with IBD and may open new scenarios for psychodynamic approaches to the treatment of the emotional disturbances in IBD based on attachment and mentalization theory.

## Figures and Tables

**Table 1:** Sociodemographic and clinical characteristics of the two study groups. Values denote mean (±standard deviation) or numbers of subjects.

	IBD patients *N* = 96	Healthy controls *N* = 102	*p*
Sociodemographics			
Age	39.64 (±14.06)	36.30 (±12.29)	n.s.
Sex	48 female, 48 male	50 female, 52 male	n.s.
Education (years)	13.41 (±3.09)	14.07 (±2.12)	n.s.
Clinical characteristics			
Disease	64 CD, 32 UC		
Disease duration (years)	12.39 (±11.44)		
Age at diagnosis (years)	27.56 (±12.09)		
Disease activity (CDAI and PMS)	55 remission		
	22 mild activity		
	19 moderate activity		
Therapy	19 no treatments		
	29 5-ASA		
	3 azathioprine		
	5 corticosteroids		
	40 biologic agents		
Surgery	75 never treated		
	21 previously treated		
Psychometric measures			
Eyes test	22.21 (±4.38)	24.37 (±3.17)	<.001
	Male 21.93 (±4.63)		
	Female 22.48 (±4.14)		n.s.
		Male 23.47 (±3.59)	
		Female 25.15 (±2.55)	.012
ASQ Confidence	30.46 (±5.52)	31.97 (±4.37)	n.s.
ASQ Discomfort with closeness	35.92 (±6.64)	34.76 (±5.47)	n.s.
ASQ Relationships as secondary	15.30 (±6.65)	15.22 (±4.97)	n.s.
ASQ Need for approval	20.73 (±5.98)	20.68 (±5.11)	n.s.
ASQ Preoccupation with relationships	28.76 (±6.65)	26.73 (±5.18)	.034
RFQ certainty	1.12 (±.99)	.99 (±.63)	n.s.
RFQ uncertainty	.72 (±.62)	.59 (±.49)	n.s.

**Table 2:** Correlation analyses in the healthy control group.

	RFQ certainty	RFQ uncertainty	ASQ Confidence	ASQ Discomfort with closeness	ASQ Relationships as secondary	ASQ Need for approval	ASQ Preoccupation with relationships
Eyes test	.056	.02	.76	-.019	-.12	.15	.01
RFQ certainty		-.566^∗∗∗^	.092	-.275^∗∗^	-.043	-.234^∗^	-.377^∗∗∗^
RFQ uncertainty			-.167	.159	.117	.252^∗^	.300^∗∗^
ASQ Confidence				-.284^∗∗^	-.114	-.180	-.071
ASQ Discomfort with closeness					.454^∗∗∗^	.332^∗∗^	.336^∗∗∗^
ASQ Relationships as secondary						.246	.202^∗^
ASQ Need for approval							.512^∗∗∗^

^∗^ *p* < .05; ^∗∗^*p* < .01; ^∗∗∗^*p* < .001. RFQ = reflective functioning questionnaire; ASQ = attachment style questionnaire.

**Table 3:** Correlation analyses in the IBD patient group.

	RFQ certainty	RFQ uncertainty	ASQ Confidence	ASQ Discomfort with closeness	ASQ Relationships as secondary	ASQ Need for approval	ASQ Preoccupation with relationships	Disease activity	Disease duration
Eyes test	.056	-.049	-.163	.007	-.295^∗∗^	-.045	-.074	-.327^∗∗^	-.083
RFQ certainty		-.478^∗∗∗^	.120	-.058	.004	-.195	-.137	.067	.062
RFQ uncertainty			.020	.021	.012	.243^∗^	.267^∗∗^	.023	-.037
ASQ Confidence				-.182	-.043	-.098	-.214^∗^	.149	.078
ASQ Discomfort with closeness					.295^∗∗^	.417^∗∗^	.244^∗^	-.017	-.030
ASQ Relationships as secondary						.356^∗∗∗^	.137	.077	.154
ASQ Need for approval							.518^∗∗∗^	-.203	-.020
ASQ Preoccupation with relationships								-.113	-.217^∗^

^∗^ *p* < .05; ^∗∗^*p* < .01; ^∗∗∗^*p* < .001. IBD = inflammatory bowel disease; RFQ = reflective functioning questionnaire; ASQ = attachment style questionnaire. The disease activity was measured using Crohn's disease activity index (CDAI) for CD and the Partial Mayo Score (PMS) for UC.

## Data Availability

The psychometric data used to support the findings of this study are available from the corresponding author upon request.
